# Comparative analysis of metamorphopsia and aniseikonia after vitrectomy for epiretinal membrane, macular hole, or rhegmatogenous retinal detachment

**DOI:** 10.1371/journal.pone.0232758

**Published:** 2020-05-08

**Authors:** Hisashi Fukuyama, Hiroto Ishikawa, Yuki Komuku, Takashi Araki, Naoki Kimura, Fumi Gomi

**Affiliations:** Department of Ophthalmology, Hyogo College of Medicine, Nishinomiya, Japan; University of Warmia, POLAND

## Abstract

This study investigated postoperative changes in metamorphopsia and aniseikonia in eyes that underwent vitrectomy for epiretinal membrane (ERM), macular hole (MH), or rhegmatogenous retinal detachment (RRD). In total, 166 eyes were included from 166 patients with ERM, MH, or RRD who underwent primary vitrectomy. Metamorphopsia and aniseikonia were quantified by M-CHARTS and the New Aniseikonia Test (NAT). Best-corrected visual acuity (BCVA), M-CHARTS, NAT assessments, and OCT examination were performed at 1, 3, and 6 months postoperatively. Of the 166 eyes, 65 had ERM, 21 had MH, 42 had macula-off RRD, and 38 had macula-on RRD. BCVA improved significantly between 1 and 6 months postoperatively in eyes with ERM, MH, and macula-off RRD (P = 0.0057, P = 0.0065, and P = 0.0021, respectively). M-CHARTS scores at 1 month postoperatively significantly decreased in eyes with ERM (P = 0.0034) and tended to decrease in eyes with MH (P = 0.068). NAT scores did not change between baseline and 1 month postoperatively in eyes with ERM or MH. Between 1 and 6 months postoperatively, M-CHARTS and NAT scores significantly decreased in eyes with macula-off RRD (P = 0.0064 and P = 0.0009, respectively), but not in eyes with ERM, MH, or macula-on RRD. At 6 months postoperatively, significant metamorphopsia was evident in 33.3% of eyes with ERM, 29.2% of eyes with MH, and 35.7% of eyes with macula-off RRD; 61.5% of eyes with ERM showed macropsia and 52.3% of eyes with macula-off RRD showed micropsia. In eyes with ERM, more central retinal thickness (CRT) correlated with postoperative BCVA, and deep retinal folds on enface OCT image correlated with postoperative metamorphopsia. In eyes with macula-off RRD, less CRT correlated with postoperative BCVA, and tended to correlate with postoperative micropsia. Macular morphologies could contribute to differences in postoperative visual acuity, metamorphopsia, and aniseikonia.

## Introduction

Metamorphopsia is a symptom of distorted vision that causes deviation of either vertical or horizontal lines in various macular disorders. Metamorphopsia is considered to be caused by displacement of the retina, which results in a false localization of the image observed by displaced elements. [1,2] Aniseikonia is a binocular condition in which the image in one eye is perceived to differ in size, relative to the image in the other eye. The two types of aniseikonia are optically induced and retinally induced. [3,4]

Various vitreoretinal diseases cause metamorphopsia and aniseikonia; these diseases include epiretinal membrane (ERM), macular hole (MH), and rhegmatogenous retinal detachment (RRD). [5–12] Pars plana vitrectomy (PPV) is an effective treatment for these retinal diseases, and recent advances in PPV have led to improvements in anatomical success and postoperative visual acuity; however, metamorphopsia combined with aniseikonia often persists and causes considerable problems.

Gradual changes in metamorphopsia and aniseikonia, and their relationships with visual acuity after PPV, have been observed in eyes with ERM [10], MH [12], and RRD [11,13,14]; however, there have been no comparative reports of the characteristics of visual symptoms among these diseases. Because each disease affects retinal morphology in a different manner, a direct comparison would provide meaningful information regarding the relationship between visual function and morphological changes. The present study was performed to compare postoperative changes in metamorphopsia and aniseikonia among eyes with ERM, eyes with MH, and eyes with RRD.

## Participants and methods

### Patients

This was a retrospective study of consecutive patients with unilateral ERM, patients with MH, and patients with RRD, who were followed up for >6 months after PPV at Hyogo College of Medicine between October 2016 and April 2018. The Institutional Review Board of Hyogo College of Medicine approved this study (approval no. 3334), which was conducted in accordance with the guidelines of the Declaration of Helsinki. The requirement for written informed consent was waived by the ethics committee due to the retrospective and observational nature of the study. The data in this study were anonymized before use in analyses.

Exclusion criteria included a previous history of vitreoretinal surgery, preexisting macular conditions (e.g., age-related macular degeneration, vascular occlusive disease, and/or diabetic retinopathy), presence of complicated vitreoretinal diseases (e.g., proliferative vitreoretinopathy Grade C, RRD with MH, and/or RRD with ERM), requirement of additional PPV during the follow-up period, presence of anisometropia >2.0 diopters before and/or after surgery, presence of myopia >10.0 diopters, and/or postoperative visual acuity >1.0 logarithm of minimum angle of resolution.

### Clinical examinations

Best-corrected visual acuity (BCVA) measurements, metamorphopsia and aniseikonia assessments, and optical coherence tomography (OCT) examinations were performed at baseline and at 1, 3, and 6 months after vitrectomy in eyes with ERM and eyes with MH. In eyes with RRD, no preoperative metamorphopsia and aniseikonia assessments were performed, nor were enface OCT examinations performed. BCVA measurements were performed using Landolt C charts. The degree of metamorphopsia was quantified by M-CHARTS (Inami, Tokyo, Japan); the degree of aniseikonia was quantified by the New Aniseikonia Test (NAT; Handaya, Tokyo, Japan).

The M-CHARTS examination was performed for vertical lines and then rotated 90 degrees; the same test was performed for horizontal lines at a distance of 30 cm with refractive correction. After all vertical and horizontal M-CHARTS scores had been obtained, mean values of vertical and horizontal examination scores were used for data analysis (M-score). In this study, an M-score of ≥0.5 was regarded as significant metamorphopsia; an M-score between 0.2 and 0.5 was regarded as mild metamorphopsia. [9]

In the NAT examination, each patient wore a red filter on the affected eye and a green filter on the normal fellow eye; they then compared the sizes of pairs of red and green semicircles on clinical charts. Measurements were performed in vertical meridians and horizontal meridians at a distance of 40 cm with refractive correction; mean values of the recorded measurements were used for analysis (NAT score). In this study, the magnitude of aniseikonia was expressed as a percentage; a positive number was indicative of macropsia, while a negative number was indicative of micropsia. Aniseikonia of ≥2% was defined as macropsia, whereas aniseikonia of <-2% was micropsia. Both M-CHART and NAT examinations were performed by professional orthoptists who were blinded to the fundus findings of the patients in this study.

The OCT examination included cross-sectional images obtained by spectral-domain OCT (Spectralis, Heidelberg Engineering, Heidelberg, Germany) and enface OCT images obtained by swept-source (SS)-OCT (DRI-OCT Triton, Topcon Corp., Tokyo, Japan). Cross-sectional OCT images were centered on the fovea and enface OCT images covered a 6 × 6-mm square area, including the macula.

Central retinal thickness (CRT) was measured manually using B-scan OCT in the foveal center. In eyes with macula-off RRD and eyes with MH, CRT was not measured preoperatively. In addition to measurement of CRT, qualitative analyses were performed in three retinal slabs (i.e., inner, middle, and outer) on enface OCT. In this study “inner retina” was the region from the internal limiting membrane (ILM) to the border between the inner plexiform layer (IPL) and inner nuclear layer (INL); “middle retina” was the region from the border of the IPL and INL to 91.0 μm from the retinal pigment membrane (RPE); and “outer retina” was the region from 91.0 μm from the RPE to the RPE, including outer retinal bands. [15] The location of each layer was based on automated layer segmentation performed by swept-source-OCT instrument software (IMAGEnet 6, software version 1.21; Topcon Corp.).

### Surgical techniques

Twenty-five or 27-gauge PPV was performed in all eyes using a vitrectomy system (Constellation; Alcon Laboratories, Fort Worth, TX, USA). Simultaneous cataract surgery was performed in eyes with cataracts of grade-2 nuclear sclerosis or cortical opacity. No eyes underwent scleral buckling procedures or received perfluorocarbon liquid. In eyes with ERM and eyes with MH, ERM and/or ILM was removed in an area of 2–3 disc diameter around the macula with intraocular forceps. Brilliant Blue G solution (0.1–0.2 ml, 0.025%) was applied to the macular area. For eyes with MH and eyes with RRD, 20% sulfur hexafluoride gas was used as internal tamponade.

### Statistical analysis

Decimal BCVA values were converted to the logarithm of minimum angle of resolution units for statistical analysis. In accordance with a previously reported method, counting fingers and hand motion were set to 2.0 and 2.30 logarithm of minimum angle of resolution units, respectively. [16] All statistical analyses were performed by means of *EZR* software (Saitama Medical Center and Jichi Medical University). [17] Data were expressed as means ± standard deviations. The Wilcoxon signed-rank test was used to compare preoperative and postoperative BCVA values, M-scores, and NAT scores, CRT. The Friedman test was used to assess changes in BCVA values, M-scores, and NAT scores. When a significant difference was detected, the Bonferroni post hoc test for multiple comparisons was conducted to identify time points with significant differences. Correlations among BCVA values, M-scores, and NAT scores were determined by *Spearman rank correlation* coefficient. P < 0.05 was considered statistically significant. Fisher’s exact test and the Kruskal–Wallis test were used to compare differences among eyes with ERM, eyes with MH, eyes with macula-off RRD, and eyes with macula-on RRD.

## Results

In total, 357 eyes of 357 consecutive patients underwent primary vitrectomy for ERM, MH, or RRD during the study period. Eyes were excluded for the following reasons: preexisting macular conditions (40 eyes), proliferative vitreoretinopathy Grade C (three eyes), anisometropia >2.0 diopters (36 eyes), myopia >10.0 diopters (23 eyes), second PPV during the study period (16 eyes), and follow-up <6 months (73 eyes). After application of exclusion criteria, 166 eyes of 166 patients were included in the study. Table 1 summarizes the clinical characteristics of included patients.

**Table 1:** Preoperative clinical characteristics of patients with epiretinal membrane, macular hole, and rhegmatogenous retinal detachment.

	Total	ERM	MH	M-off RRD	M-on RRD	P
**Number of patients (eyes)**	166 (166)	65 (65)	21 (21)	42 (42)	38 (38)	
**Age (years)**	63.8 ± 11.1 (19–91)	69.8 ± 8.4 (46–87)	66.7 ± 9.9 (45–91)	58.7 ± 10.0 (42–80)	57.0 ± 11.3 (19–79)	< 0.0001^a^
**Sex (male/female)**	76/90	29/36	4/17	23/19	20/18	0.040^b^
**Eye (right/left)**	85/81	35/30	13/8	20/22	17/21	0.577^b^
**Preoperative lens status (phakic/pseudophakic)**	136/30	50/15	18/3	34/8	34/4	0.452^b^

Abbreviations: ERM = epiretinal membrane, MH = macular hole, RRD = rhegmatogenous retinal detachment, M-off = macula off, M-on = macula on.

^a^ P was calculated using the Kruskal–Wallis test

^b^ P was calculated using Fisher’s exact test

Of the 166 eyes, 65 had ERM, 21 had MH, and 80 had RRD. Of the eyes with RRD, 42 (51.0%) had macula-off RRD (defined as RRD involving the fovea) and 38 had macula-on RRD. One hundred twelve eyes (67.5%) underwent phacoemulsification combined with PPV. Postoperative absolute differences in spherical equivalent between the two eyes were not significantly different: 0.70 ± 0.56 at month 1 and 0.72 ± 0.63 at month 6 (P = 0.23 and P = 0.25, respectively).

### Time course of BCVA values, M-scores, and NAT scores

**Fig 1:**
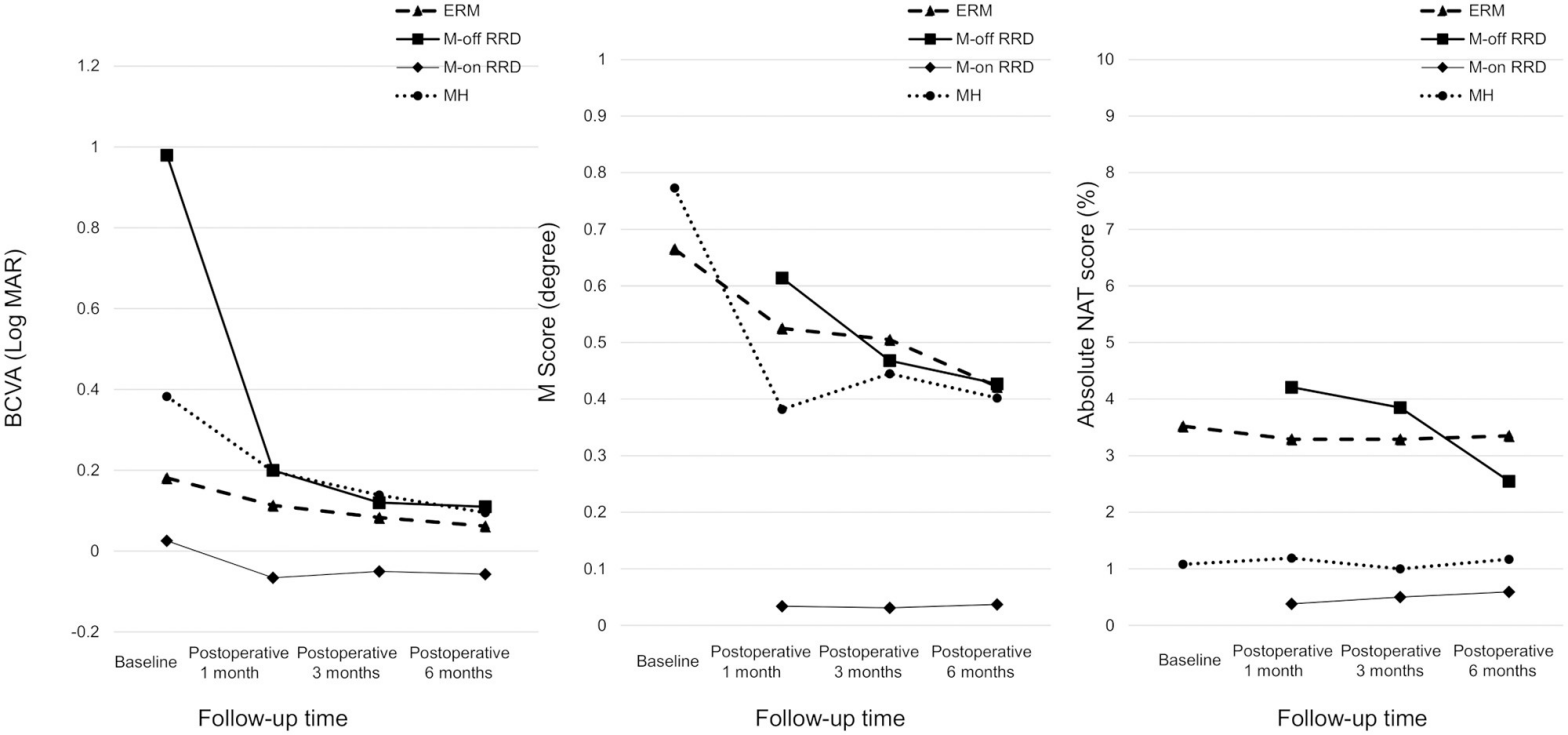
Time Course of Best-Corrected Visual Acuity (BCVA), M-score, and absolute NAT score. Abbreviations: ERM = epiretinal membrane, MH = macular hole, RRD = rhegmatogenous retinal detachment, M-off = macula off, M-on = macula on.

Fig 1 shows gradual changes in BCVA values, M-scores, and NAT scores. For eyes with macula-on RRD, there were no significant differences in BCVA values, M-scores, or absolute NAT scores throughout the follow-up period.

BCVA at 1 month postoperatively was significantly better, compared with preoperative values, for eyes with ERM, eyes with MH, and eyes with macula-off RRD (P = 0.0036, P = 0.0009, and P < 0.0001, respectively). Furthermore, BCVA at 6 months postoperatively was significantly better, compared with BCVA at 1 month postoperatively, for eyes with ERM, eyes with MH, and eyes with macula-off RRD (P = 0.0057, P = 0.0065, and P = 0.0021 respectively). BCVA values at 6 months postoperatively were 0.062 ± 0.12 (ERM), 0.095 ± 0.12 (MH), and 0.11 ± 0.21 (macula-off RRD); these values did not significantly differ among the three groups (P = 0.31).

At baseline, the mean vertical M-CHARTS (VM) scores were 0.65 ± 0.54 (ERM) and 0.86 ± 0.61 (MH), while the mean horizontal M-CHARTS (HM) scores were 0.68 ± 0.60 (ERM) and 0.69 ± 0.61 (MH), respectively. Notably, VM and HM scores did not significantly differ between eyes with ERM (P = 0.92) and those with MH (P = 0.182). M-scores at baseline were 0.66 ± 0.51 (ERM) and 0.77 ± 0.60 (MH); these also did not significantly differ between groups of eyes (P = 0.65).

M-scores at 1 month postoperatively were significantly better than baseline in eyes with ERM (P = 0.0034), and tended to be better than baseline in eyes with MH (P = 0.068). M-scores did not significantly differ between 1 month and 6 months postoperatively in eyes with ERM, eyes with MH, and eyes with macula-on RRD. In contrast, M-scores in eyes with macula-off RRD at 3 and 6 months postoperatively were significantly better than at 1 month postoperatively (P = 0.041 and P = 0.0064, respectively). At 6 months postoperatively, M-scores were 0.42 ± 0.44 in eyes with ERM (mean VM scores = 0.48 ± 0.51, mean HM scores = 0.36 ± 0.51), 0.40 ± 0.37 in eyes with MH (mean VM scores = 0.40 ± 0.39, mean HM scores = 0.40 ± 0.42), and 0.43 ± 0.37 in eyes with macula-off RRD (mean VM scores = 0.48 ± 0.41, mean HM scores = 0.38 ± 0.37); these values did not significantly differ among the three groups (P = 0.83).

Regarding absolute NAT scores, there were no significant differences between before and after PPV, as well as between 1 and 6 months postoperatively, in eyes with ERM and eyes with MH. Conversely, absolute NAT scores at 6 months postoperatively significantly improved, compared with those at 1 month, in eyes with macula-off RRD (P = 0.0009). Absolute NAT scores at 6 months postoperatively were 3.35 ± 3.13 (ERM), 1.17 ± 1.64 (MH), and 2.55 ± 2.58 (macula-off RRD); scores were lower in eyes with MH than in eyes with ERM (P = 0.0022) and in eyes with macula-off RRD (P = 0.065).

### Proportions of eyes with metamorphopsia and aniseikonia at 6 months postoperatively

**Fig 2:**
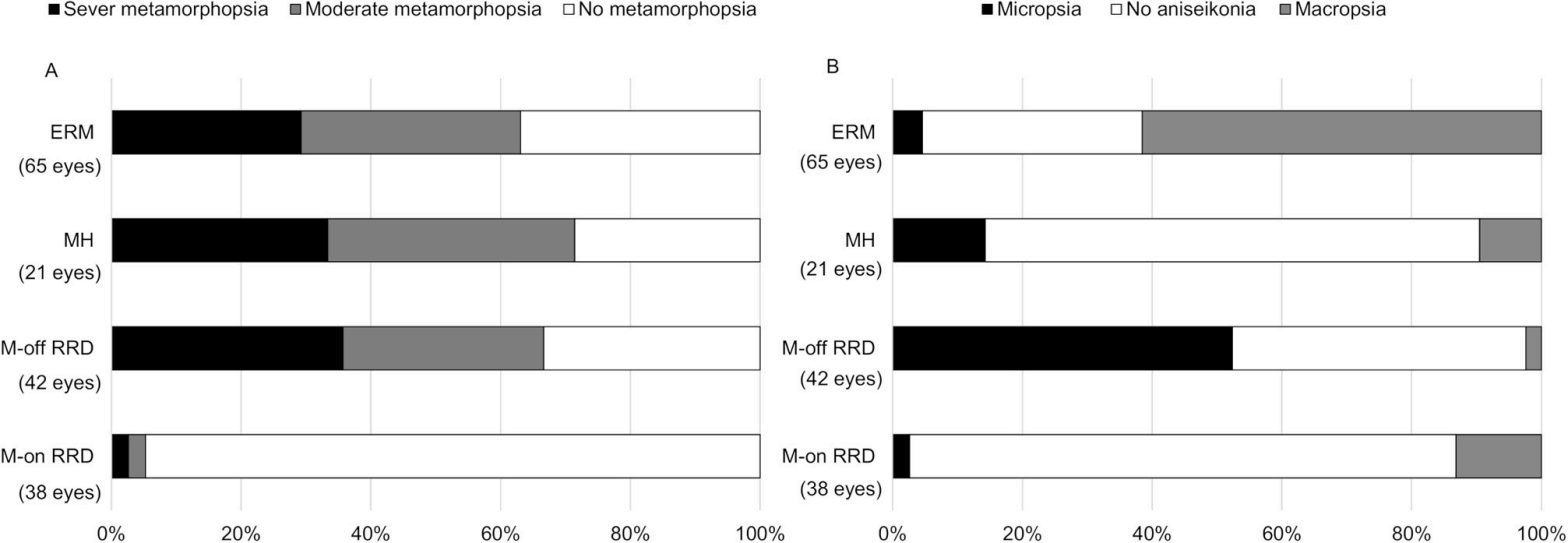
Distributions of metamorphopsia and aniseikonia at 6 months postoperatively. Abbreviations: ERM = epiretinal membrane, MH = macular hole, RRD = rhegmatogenous retinal detachment, M-off = macula off, M-on = macula on.

Fig 2 shows the proportions of eyes with metamorphopsia and aniseikonia at 6 months postoperatively, compared among the diseases. The proportions of eyes with ERM, eyes with MH, eyes with macula-off RRD, and eyes with macula-on RRD that exhibited significant metamorphopsia were 33.3%, 29.2%, 35.7%, and 2.6%, respectively (Fig 2A). NAT scores at 6 months postoperatively ranged from -12% to 14.5%. Of the eyes with ERM, 61.5% had macropsia; of the eyes with macula-off RRD, 52.3% had micropsia. Of the eyes with MH and eyes with macula-on RRD, no aniseikonia was observed in 76.2% and 84.2%, respectively (Fig 2B).

### Retinal morphological changes on OCT

Table 2 shows CRT at baseline and postoperatively. In eyes with ERM, CRT at 6 months postoperatively was significantly thinner than CRT at 1 month postoperatively. Conversely, eyes with macula-on RRD exhibited significantly thicker CRT at 6 months postoperatively than at 1 month postoperatively. CRT at 6 months postoperatively was significantly thicker in eyes with ERM than in eyes with MH, eyes with macula-off RRD, and eyes with macula-on RRD (all P < 0.001).

**Table 2:** Changes in central retinal thickness.

	Baseline	1 month postoperatively	6 months postoperatively	P
**ERM**	378 ± 130^a^	359 ± 95	334 ± 87	< 0.0001
**MH**		240 ± 53	235 ± 56	0.376
**M-off RRD**		205 ± 47	210 ± 48	0.15
**M-on RRD**	208 ± 24	215 ± 25	234 ± 39	< 0.0001

Abbreviations: ERM = epiretinal membrane, MH = macular hole, RRD = rhegmatogenous retinal detachment, M-off = macula off, M-on = macula on.

^a^All values are shown in μm.

Baseline and postoperative morphological characteristics on enface images are summarized in Table 3. At baseline, enface images of the inner retina showed folds caused by ERM in eyes with ERM and eyes with MH; postoperatively, those eyes exhibited dissociated nerve fiber layer, separated from the fovea. A few eyes with RRD showed folds associated with secondary ERM.

**Table 3:** Retinal morphological changes on optical coherence tomography.

	Baseline			1 month postoperatively			6 months postoperatively		
	Inner retina	Middle retina	Outer retina	Inner retina	Middle retina	Outer retina	Inner retina	Middle retina	Outer retina
**ERM eyes (%)**	Radiating folds due to ERM 65 (100.0)	Cyst 10 (15.4) Folds 44 (67.7) None 11 (16.9)	None 65 (100.0)	DONFL 3 (4.6) Cyst 1 (1.5) None 61 (93.8)	Cyst 11 (16.9) Folds 17 (26.2) None 37 (56.9)	None 65 (100.0)	DONFL 16 (24.6) None 49 (75.4)	Cyst 7 (10.8) Folds 12 (18.5) None 46 (70.8)	None 65 (100.0)
**MH eyes (%)**	Radiating folds due to ERM 6 (28.6) None 15 (71.4)	Cyst 20 (95.2) None 1 (4.8)	MH 21 (100.0)	DONFL 6 (28.6) None 15 (71.4)	Cyst 4 (19.0) None 17 (81.0)	Foveal detachment 12 (57.1) None 9 (42.9)	DONFL 16 (76.2) None 5 (23.8)	Cyst 5 (23.8) None 16 (76.2)	Foveal detachment 2 (95.2) None 19 (4.8)
**M-off RRD eyes (%)**				Radiating folds due to ERM 4 (9.5) None 38 (90.5)	Cyst 1 (2.4) None 41 (97.6)	Folds 42 (100.0)	Radiating folds due to ERM 5 (11.9) None 37 (88.1)	Cyst 4 (9.5) None 38 (90.5)	Folds 42 (100.0)
**M-on RRD eyes (%)**				Radiating folds due to ERM 1 (2.6) None 37 (97.4)	None 38 (100.0)	None 38 (100.0)	Radiating folds due to ERM 3 (7.9) None 35 (92.1)	Cyst 1 (2.6) None 37 (97.4)	None 38 (100.0)

Abbreviations: ERM = epiretinal membrane, MH = macular hole, RRD = rhegmatogenous retinal detachment, M-off = macula off, M-on = macula on, DONFL = dissociated nerve fiber layer

**Fig 3:**
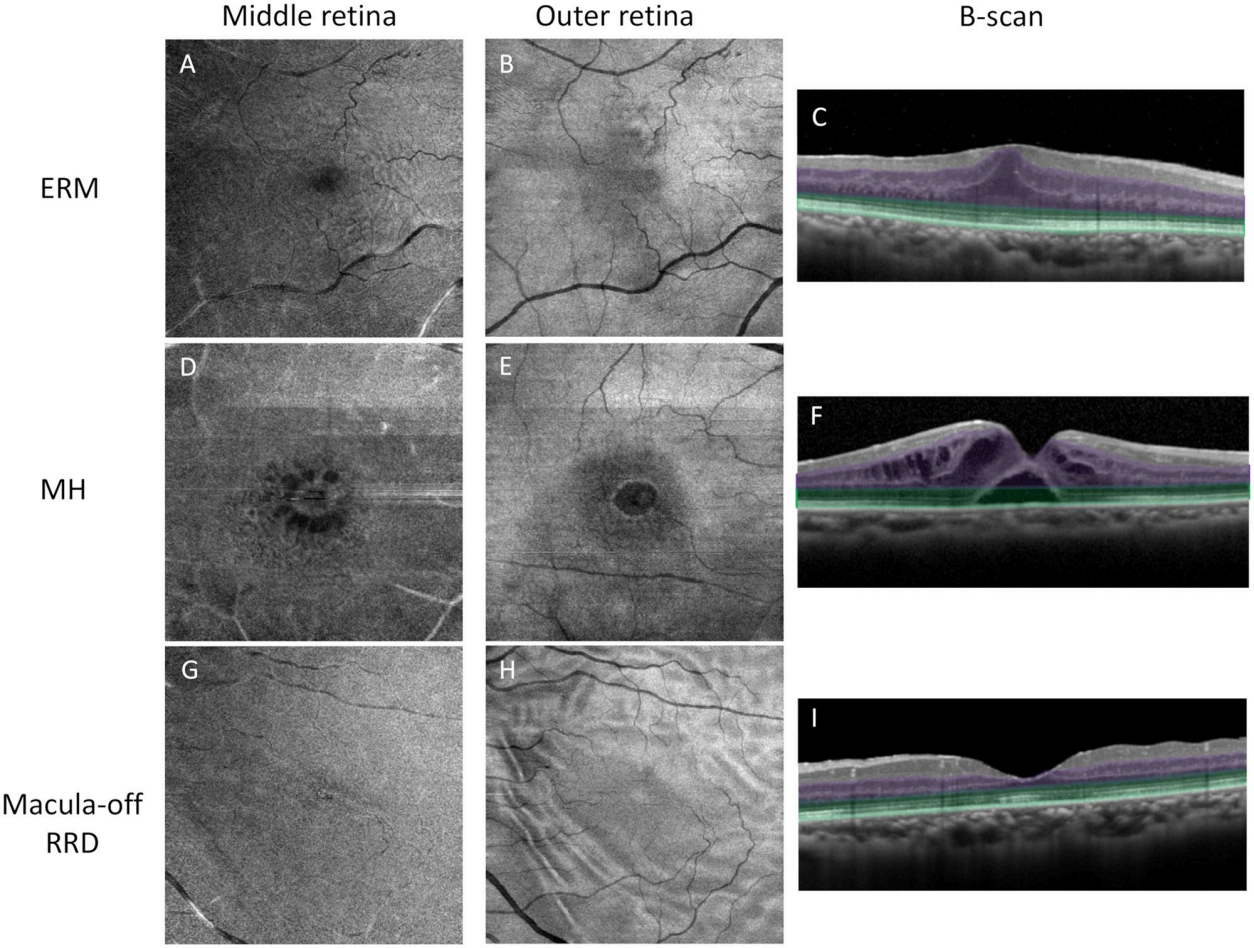
Macular morphological changes on enface OCT findings. Enface OCT in the middle retina (A, D, G) and outer retina (B, E, H), as well as cross-sectional OCT images (C, F, I) for eyes with ERM (A–C), eyes with MH (D–F), and eyes with macula-off RRD (G–I). Middle retina comprises the area between the border of the inner plexiform layer and inner nuclear layer, extending to 91.0 μm from the RPE (highlighted in purple on cross-sectional OCT); outer retina comprises the area between 91.0 μm from the RPE to the RPE (highlighted in green) (C, F, I). In eyes with ERM, undulation of the outer nuclear layer appeared as concentric folds on en face OCT images of middle retina (A); in eyes with MH, retinal cysts (D) were observed in middle retina, while foveal detachment was observed in outer retina (E). In eyes with macula-off RRD, en face OCT images of outer retina showed several sharp and dull folds (H). Abbreviations: ERM = epiretinal membrane, MH = macular hole, RRD = rhegmatogenous retinal detachment, RPE = retinal pigment membrane, OCT = optical coherence tomography.

Enface images of the middle retina in eyes with ERM showed concentric fine folds (Fig 3A) in 44 eyes (67.7%) and retinal cysts (Fig 3D) in 10 eyes (15.4%) at baseline. At 6 months postoperatively, 12 eyes (18.5%) and seven eyes (10.8%) showed folds and cysts, respectively; the proportions of eyes that showed concentric folds were significantly reduced, compared with baseline (P < 0.0001).

In eyes with MH, en face OCT images of middle retina showed retinal cysts in 20 eyes (95.2%). At 6 months postoperatively, retinal cysts were observed in five eyes (23.8%). In outer retina, foveal detachment (Fig 3E) was observed in two eyes (9.5%). In eyes with macula-off RRD, retinal cysts were observed in four eyes (9.5%) in middle retina at 6 months postoperatively; folds were observed in outer retina in all eyes (Fig 3H).

### Relationships among metamorphopsia, aniseikonia, BCVA, and OCT findings

At 6 months postoperatively, neither M-scores nor absolute NAT scores were significantly correlated with BCVA in eyes with ERM or MH. A significant correlation was observed between M-scores and absolute NAT scores at 6 months postoperatively in eyes with ERM (r = 0.459, P = 0.0029). In eyes with macula-off RRD, M-scores and absolute NAT scores at 6 months postoperatively were both significantly correlated with BCVA at 6 months postoperatively (r = 0.34, P = 0.028, and r = 0.32, P = 0.039, respectively).

In eyes with ERM, CRT values at baseline and 6 months postoperatively were significantly correlated with M-scores at 6 months postoperatively (r = 0.311, P = 0.021; r = 0.289, P = 0.031, respectively); however, CRT values were not correlated with NAT scores or BCVA values at any time point. In eyes with macula-off RRD, CRT values at 6 months postoperatively were significantly correlated with BCVA at 6 months postoperatively (r = -0.371, P = 0.017) and tended to be correlated with NAT scores (r = 0.286, P = 0.070). In eyes with MH and eyes with macula-on RRD, CRT values were not significantly associated with BCVA values, M-scores, or NAT scores.

Regarding changes on enface OCT, eyes with ERM that exhibited concentric folds in middle retina at baseline showed significantly higher M-scores and NAT scores at 1 month postoperatively, compared with eyes without folds (P = 0.007 and P = 0.040, respectively); however, there were no significant associations between folds and M-scores, or between folds and NAT scores, at 6 months postoperatively (P = 0.14 and P = 0.18, respectively). In eyes with macula-off RRD, folds in outer retina remained in all eyes; however, 13 of 42 eyes (31.0%) did not exhibit metamorphopsia. In eyes with MH and eyes with macula-on RRD, morphological changes on enface OCT images were not significantly associated with BCVA values, M-scores, or NAT scores.

## Discussion

In the present study, we compared changes in metamorphopsia and aniseikonia among eyes that had undergone PPV for ERM, MH, or RRD, and investigated the relationships of these changes with visual acuity. These diseases result in distinct macular morphologies; accordingly, the current results revealed characteristic differences that were related to preoperative macular conditions.

In our study, significant metamorphopsia persisted in approximately 30% of, eyes with ERM, eyes with MH, and eyes with macula-off RRD; however, it seldom occurred in eyes with macula-on RRD. The mean M-CHARTS scores at 6 months postoperatively were 0.42 (ERM), 0.40 (MH), and 0.43 (macula-off RRD). These scores were similar to those of a previous study. [18–20] In addition, both vertical and horizontal metamorphopsia did not differ among eyes with ERM, eyes with MH, and eyes with macula-off RRD. Furthermore, 61.5% of eyes with ERM had macropsia and 52.3% of eyes with macula-off RRD had micropsia in our study; these findings are consistent with the results in a previous report by Okamoto et al., in which 68% of eyes with ERM had macropsia, and approximately half of the eyes with macula-off RRD had micropsia. [21]

BCVA showed gradual but significant improvement after PPV in eyes with ERM, eyes with MH, and eyes with macula-off RRD. In contrast, significant reductions in M-scores and absolute NAT scores were observed in eyes with macula-off RRD. In eyes with macula-off RRD, M-scores and absolute NAT scores at 6 months postoperatively were significantly correlated with BCVA. Conversely, in eyes with ERM and eyes with MH, postoperative M-scores and absolute NAT scores were not significantly correlated with BCVA scores.

Previous studies showed that outer retinal microstructure changes gradually improved during the postoperative period; moreover, postoperative visual acuity was associated with photoreceptor regeneration in eyes with ERM, eyes with MH, and eyes with RRD. [22–29] With respect to postoperative metamorphopsia and aniseikonia, several studies demonstrated no correlations with photoreceptor status; however, they revealed correlations with other factors, such as INL thickness, which suggested inner retinal displacement. [14,21,30] The prior results suggested that postoperative recovery of visual function was mainly dependent on affected retinal areas caused by different pathologies. To test this hypothesis, we assessed retinal morphological changes using OCT images. Postoperative OCT images revealed gradual resolution of retinal abnormalities, but the specific retinal characteristics differed among diseases. For example, in eyes with ERM, retinal surface folds were present due to ERM; enface OCT images of middle retina also showed concentric fine folds in 44 of 65 eyes (67.7%) and cystic changes in 10 eyes (15.4%) at baseline, as well as 12 of 65 eyes (18.5%) and seven of 65 eyes (10.8%) at 6 months postoperatively, respectively. In eyes with MH, the proportion of eyes that exhibited cystic changes in middle retina decreased from 20 of 21 (95.2%) at baseline to five of 21 (23.8%) at 6 months postoperatively; two eyes showed foveal detachment in outer retina at 6 months postoperatively. In eyes with macula-off RRD, postoperative fold-like changes were observed only in outer retina. These distinct macular morphologies could have contributed to differences in postoperative visual acuity, metamorphopsia, and aniseikonia.

The mechanism of metamorphopsia is presumably lateral photoreceptor displacement in the macula, as well as inner retinal displacement. [31] In this study, approximately one-third each of eyes with ERM (29.2%), eyes with MH (33.3%), eyes with and macula-off RRD (35.7%) showed significant metamorphopsia at 6 months postoperatively; conversely, few eyes with macula-on RRD (2.6%) showed significant metamorphopsia at this stage of follow-up. In eyes with macula-off RRD, we previously reported that findings of outer retinal folds and disrupted outer retinal bands on en face OCT were correlated with postoperative metamorphopsia. [26] In contrast, eyes with ERM exhibit dislocated retinal neurons including Müller cells, which display wave-guide coupling characteristics and light-guiding capability; [32] this type of dislocation might have a considerable impact on postoperative metamorphopsia. In the present study, postoperative metamorphopsia in eyes with ERM was significantly associated with CRT and findings of concentric fine folds in middle retina; both thickened retina and deep retinal folds suggest dislocation of retinal neurons. In eyes with MH, disruption of retinal neurons might contribute to significant postoperative metamorphopsia.

With respect to aniseikonia, altered photoreceptor distribution has been suggested as a contributing factor for retinally induced aniseikonia. [5] In the present study, a majority of eyes with ERM (61.5%) exhibited macropsia, while approximately half of the eyes with macula-off RRD (52.3%) exhibited micropsia; conversely, most of the eyes with macula-on RRD (84.2%) and eyes with MH (76.2%) exhibited no aniseikonia. In eyes with macula-off RRD, reduced photoreceptor density reportedly occurs through increased spacing between photoreceptors, which results in micropsia. [33] In contrast, mechanical distortion of retinal structure can lead to inner retinal compressions and a relative increase in the number of stimulated photoreceptors, as suggested by the findings of increased CRT and presence of concentric middle retinal folds, thereby causing macropsia in eyes with ERM.

Previous studies have found that eyes with MH tend to show micropsia because MH formation can occur when photoreceptors are stretched apart; however, after treatment, affected eyes were often symptom-free. [12,31] Similarly, most eyes with MH (76.2%) showed no aniseikonia at 6 months postoperatively in the present study.

The main limitation of the present study was its short-term follow-up period. Previous studies have shown that visual acuity, metamorphopsia, and aniseikonia improve over a duration of several years in eyes with ERM, eyes with MH, and eyes with RRD. [9,34–36] Other limitations of the study were its relatively small sample size and the retrospective design. Nevertheless, the present study revealed differences in postoperative visual function changes in eyes with distinct macular morphologies.

In conclusion, metamorphopsia and aniseikonia after PPV tended to exhibit distinct characteristics in eyes with ERM, eyes with MH, and eyes with RRD. The differences observed were presumably related to the region of the retina that was substantially stretched or distorted in the pathologic process.

## Supporting information

S1 File Original data used for analysis in this study. (XLSX)
